# 0078. Establishing a detailed short-term rat model of partial ischaemia/reperfusion injury

**DOI:** 10.1186/2197-425X-2-S1-P1

**Published:** 2014-09-26

**Authors:** G Sabbatini, A Dyson, M Singer

**Affiliations:** University College London, Bloomsbury Institute of Intensive Care Medicine, London, UK

## Introduction

Liver ischaemia/reperfusion (I/R) injury may be observed after major hepatic surgery or resuscitation from severe trauma/haemorrhage. Well-characterised and representative animal models are needed to better understand mechanisms of injury, apply effective treatments and prevent complications.

## Objectives

To establish a well-characterized rat model of partial liver I/R injury.

## Methods

Under isoflurane anaesthesia, tracheotomized male Wistar rats underwent left common carotid artery and right jugular vein instrumentation for BP measurement/blood sampling and fluid infusion (10 ml/kg/hr), respectively, and bladder catheterization for urine output measurement. Via a transverse subcostal laparotomy, blood vessels to the median and left liver lobes were occluded with a surgical clamp for 60 mins. On release of the clamp, the remaining liver lobes were ligated to prevent a steal phenomenon. The animals were observed for a further 5 hours. Sham animals underwent the same procedure except for vascular occlusion. Measurements were made of haemodynamics (BP, echocardiography), blood gas analysis, and biochemical and functional (Indocyanine Green plasma disappearance rate, ICG-PDR) liver function tests. A tissue PO_2_ probe (tPO_2_) (Oxford Optronix, UK) was placed in contact with liver tissue to measure hepatic tissue PO_2_. Tissue samples were taken to assess microscopic and ultrastructural injury both locally and remotely (data not shown).

## Results

See figure 1.

**Figure 1:**
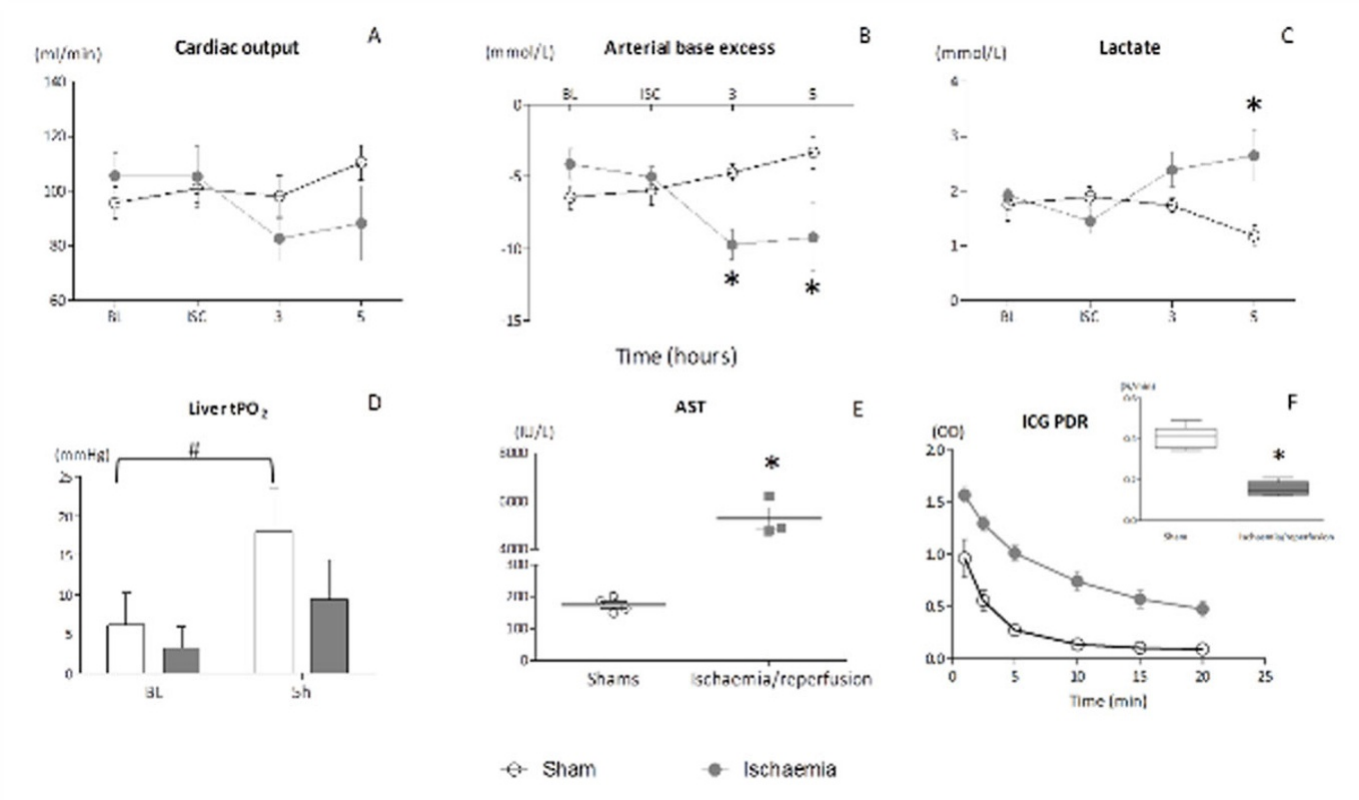
ISC= Ischaemia, BL= baseline, OD= optical density AST= aspartate aminotransferase. Data shown as mean (SEM) (A-E) or median (interquartile range) (F). Sham= 6, Ischaemia= 5/group, *p< 0.05 comparing ischaemia to sham, #p< 0.05 comparing BL to 5h. Statistics performed using t-test or two-way ANOVA and Bonferroni's test for multiple comparisons, as appropriate.

Liver ischaemia resulted in an increase of markers of hepatic injury (AST) and function (ICG-PDR and Lactate). Cardiac output was maintained but arterial base excess was different between groups. tPO_2_ at 5 hours post-reperfusion recovered in shams but not in I/R group.

## Conclusions

This severe liver I/R model demonstrates derangement of hepatic function, biochemistry, haemodynamic and ultrastructure. It offers utility for the assessment of interventions aimed at preventing or reducing I/R injury.
